# Cross-cultural adaptation and validation of the Spanish version of the Cardiopulmonary Resuscitation Motivation Scale (s-CPRMS): a cross sectional study

**DOI:** 10.1186/s12912-024-02445-3

**Published:** 2024-11-18

**Authors:** Mª Carmen Rodríguez-García, Esteban Montoya-Giménez, Helena Martínez-Puertas, José Miguel Garrido-Molina, Alba García-Viola, Verónica V. Márquez-Hernández

**Affiliations:** 1 Department of Nursing, Physiotherapy and Medicine, Faculty of Health Sciences, University of Almería, Almería, Spain; 2 Research Group CTS-1127 Epidemiology and Public Health, University of Almeria, Almería, Spain; 3 Tercio “Don Juan de Austria” 3º of La Legión, Light Infantry Brigade “Rey Alfonso XIII” II of La Legion, Almería, Spain; 4 Department of Mathematics, University of Almería, Almería, Spain; 5 Centro de Emergencias Sanitarias 061 de Almería Servicio Andaluz de Salud, Junta de Andalucía, Almeria, Spain; 6 Distrito Sanitario Almería Servicio Andaluz de Salud, Junta de Andalucía, Almeria, Spain; 7 Research Group of Health Sciences, Almería, CTS-451 Spain

**Keywords:** Cardiac arrest, Cardiopulmonary resuscitation, Cross-cultural adaptation, Motivation, Psychometric, Scale, Validation

## Abstract

**Background:**

Cardiac arrest remains a serious global health issue worldwide which claims for review and improvement. High motivation among resuscitators could lead to high-quality resuscitation and better outcomes. This study aimed to translate and cross-culturally adapt the Cardiopulmonary Resuscitation Motivation Scale into Spanish and assess the psychometric properties of the Spanish version (s-CPRMS).

**Methods:**

A sample of critical care and acuity-emergency physicians and nurses (*N* = 352) participated in an observational cross-sectional study structured in 3 phases.

**Results:**

Face and content validity was confirmed for the s-CPRMS (s-CVI = 0.88). The ordinal alpha was 0.847, ranging from 0.796 to 0.92 for the factors, indicating good reliability and temporal stability (mean ICC = 0.701; *p* < 0.001). The Exploratory Factor Analysis (EFA) results showed the s-CPRMS was distributed into four factors that explained 58% of the variance with values of the goodness of fit indices indicating an adequate fit for the model extracted in the Confirmatory Factor Analysis (CFA): χ2 (246) = 402.240; *p* < 0.001, SRMR = 0.086, RMSEA = 0.059, TLI = 0.965, CFI = 0.969, GFI = 0.990.

**Conclusions:**

The s-CPRMS is a valid and reliable instrument to assess the motivation of resuscitation teams, which might lead to potential effective strategies to resuscitation quality improvement and outcomes.

## Background

Cardiac arrest continues to be a serious global health problem worldwide [1], representing one of the leading causes of mortality despite extensive research in recent years [2–4]. The incidence of cardiac arrest is increasing globally, largely due to the rising incidence and prevalence of cardiovascular diseases; this poses a significant public health problem economically, socially, and medically, and is open to review and improvement [5, 6]. Cardiac arrest can occur both in the community (out-of-hospital cardiac arrest [OHCA]) and in the hospital (in-hospital cardiac arrest [IHCA]), potentially affecting anyone and causing everything from irreversible damage to death [7].

Immediate initiation of high-quality cardiopulmonary resuscitation (CPR), through chest compressions, defibrillation, or both, is one of the most important predictive factors to improve outcomes after cardiac arrest [8, 9]. High-quality CPR is crucial in the care of patients with cardiac arrest, associated with improved survival rates and neurological prognosis [10, 11] regardless of where it occurs [12]. Thus, the performance of high-quality CPR becomes an essential priority among healthcare professionals [13, 14]. However, chest compressions often do not meet the standards of resuscitation guidelines and are one of the main focuses of research in the continuous improvement of CPR quality in search of potentially effective strategies to improve its quality [15–17].

The relationship between motivation and learning is widely known [18–20], although little studied in CPR. Most research has studied the effects of training on CPR improvement in different study populations [21, 22], without delving into the role of motivation. The success of these training programs and the quality of CPR can be influenced by certain intrinsic factors of the resuscitators [23, 24] such as motivation [25]. In recent years, specific instruments have been designed to assess the motivation of healthcare workers in CPR, like the Cardiopulmonary Resuscitation Motivation Scale, arguing that high motivation among resuscitators could lead to high-quality CPR [26]. Also, previous research using the original CPRMS instrument has showed that nurses would be motivated to perform quality cardiopulmonary resuscitation if they had a feeling of success, with differences regarding individual’s factors such as level of education and participation in CPR workshops [27]. However, the scarce literature and the lack of cross-cultural adaptation and validation of the instrument to other cultures and contexts complicate the comparative analysis of the effectiveness of different strategies implemented to improve the quality of CPR in different countries worldwide, as well as the mediating effect of motivation on CPR outcomes.

## Methods

### Aim

This study aimed to translate and cross-culturally adapt the Cardiopulmonary Resuscitation Motivation Scale into Spanish and assess its psychometric properties.

### Design

A cross-sectional descriptive observational study was conducted for the adaptation and cross-cultural analysis of instruments through a rigorous standardized process divided into three phases: (1) adaptation and translation of the instrument into Spanish, (2) analysis of content validity and reliability in a pilot study, and (3) final analysis of construct validity and reliability of the Spanish version of the Cardiopulmonary Resuscitation Motivation Scale (s-CPRMS).

### Participants

The study was carried out in seven care units from different health centers in Spain. The potential study population consisted of doctors and nurses from critical care and emergency departments. The study sample was obtained by convenience sampling. The inclusion criteria were: (a) being a doctor and/or nurse with at least one year of work experience, (b) working in critical care and emergency departments, (c) voluntarily consenting to participate in the study and signing informed consent. The recommended minimum of 30 subjects was guaranteed for piloting [28] and between five and ten subjects per item of the instrument [29, 30] was ensured. Subjects who participated in the pilot (*N* = 50) were excluded from the sample for EFA: exploratory factor analysis (*N* = 120) and CFA: confirmatory factor analysis (*N* = 182). EFA participants were not included in the CFA.

### Data collection

Data was collected between March and May 2023 through an online self-administered questionnaire designed by the investigators. The questionnaire consisted of the following sections: (1) study information and informed consent, (2) sociodemographic characteristics, and (3) s-CPRMS. Data collection was performed between the months of March and April 2023. Potentially eligible subjects were contacted, and a link to complete the online questionnaires, with an estimated duration of 10 min, was sent. Participants were contacted four weeks later to analyze the temporal stability of the subjects’ responses through test-retest [31].

### Measurements

Sociodemographic variables such as gender, age, level of education, professional category, work experience, centre and care unit, and years of experience in critical care and emergency departments were collected. The original version of The Cardiopulmonary Resuscitation Motivation Scale (CPRMS) was developed by Assarroudi et al. [26] to measure CPR motivation using 43 items structured in eight dimensions: (1) Facilitators of resuscitation with items 38, 39, 40, 41, 42 and 43; Sense of accomplishment with items 27, 28, 29, 30, 31, 32 and 33; (3) High chance of success with items 4, 8, 12, 13, 14, 14, 15, 16 and 17; (4) Low chance of success with items 5, 6, 7, 9, 10 and 11; (5) Recognition and appreciation with items 21, 22, 23, 24, 25, 26; (6) Responsibility with items 34, 35, 36, 37; (7) Perceived importance comprises items 1, 2 and 3; and (8) Beliefs with items 18, 19 and 20. The responses use a 5-point Likert scale ranging from 1 (never) to 5 (always) for the first three items and from 1 (strongly disagree) to 5 (strongly agree) for the rest of the items. Higher scores indicate better CPR motivation. The total reliability for the instrument was α = 0.92.

### Translation and cross-cultural instrument adaptation

The translation and adaptation of the CPRMS instrument into Spanish was carried out through a rigorous process to ensure cross-cultural equivalence of content, semantic, technical, criterion, and conceptual aspects [32]. The phases of the process included (1) forward/backward translation, (2) review and feedback by translation experts, and (3) cross-cultural evaluation in terms of equivalence, relevance, and accuracy of translation for each item and the overall instrument using the Content Validity Index (CVI). translation of both each item and overall instrument using Content Validity Index (CVI). The forward translation into Spanish was carried out independently by two bilingual (Spanish-English) native Spanish translators with medical language translation experience. Later, the instrument was back-translated into English by an independent bilingual (English-Spanish) native English translator. The importance of semantic rather than literal translation was emphasized to ensure equivalence to the original version. The researchers reviewed the translation and assessed the equivalence between both versions before sending the instrument to experts for further evaluation.

### Psychometric testing

#### Face validity

Face validity was assessed by a multidisciplinary group of doctors and nurses (*N* = 10) with experience in critical care and emergency departments to determine the level of difficulty in understanding, appropriateness, and relationship with the scale’s dimensions, as well as ambiguity and risk of error with the words and wording of the items. All contributions were considered to improve the draft version and ensure the face validity of the items.

#### Content validity

Initially, the s-CPRMS consisted of 43 items, as in the original version. Content validity was analyzed by a panel expert (*N* = 13) who evaluated the content of the items in terms of relevance for measuring motivation in CPR. The expert panel consisted of a multidisciplinary team of researchers with experience in developing and validating instruments in health sciences, as well as educators with PhDs and professionals in critical care, emergency, and pre-hospital care in the Spanish health context. The CVI for the entire scale (s-CVI) and for each item (i-CVI) was calculated using a Likert scale of 1 to 4. (1 = not relevant at all, 2 = not very relevant, 3 = quite relevant, and 4 = very relevant). The researchers agreed on a minimum score for s-CVI > 0.80, as well as, the inclusion of items with i-CVI ≥ 0.78. The i-CVI was calculated by summing the scores of experts who considered the item quite relevant or very relevant divided by the total number of experts [33]. Item wording was also evaluated in terms of understanding and clarity, with the possibility of suggesting improvements through comments to ensure the suitability of the instrument to the Spanish context. Experts reported adequate equivalence, readability, and understanding without the need to add new items to the final version of the s-CPRMS, which consisted of a total of 24 items.

#### Construct validity

In this study, the construct validity of the s-CPRMS was tested by performing an exploratory factor analysis (EFA) and confirmatory factor analysis (CFA).

An EFA was computed using the principal axis factoring method with oblimin rotation to examine construct validity. In this study, data were not normally distributed and a polychoric matrix was used for EFA as the most suitable when all the variables are Likert scale [34]. The appropriateness of the data for EFA was assessed by the Kaiser–Meyer–Olkin (KMO) and Bartlett’s test of sphericity (BTS), considering appropriate to conduct an EFA when KMO > 0.70 and the BTS was significant (*p* < 0.05). The number of the factors to be extracted was determined using scree plot with parallel analysis and eigenvalues ≥ 1. A minimum factor loading ≥ 0.3 was set to retain the items in the extracted factors.

A CFA was computed to verify the latent structure of s-CPRMS and the model fit proposed by EFA. The goodness of fit indexes used for model assessment included χ2, Standardized Root Mean Square Residual (SRMR) ≤ 0.08 indicating good fit, Root Mean Square Error Approximation (RMSEA) ≤ 0.05 indicating a well-fitting model, Tucker–Lewis Index (TLI), Comparative Fit Index (CFI) and Goodness-of-Fit Index (GFI) whit values ≥ 0.95 indicating excellent fit [35].

#### Reliability

A pilot study was conducted with physicians and nurses (*N* = 50) working in critical care and emergency care services to analyze the reliability of the instrument, internal consistency and temporal stability. Cronbach’s alpha values could underestimate the results of ordinal measurement scales [36, 37]; therefore, ordinal alpha was calculated to estimate the internal consistency of the response scale of ordinal items, considering acceptable and excellent values > 0.7 and > 0.9, respectively [38]. The mean inter-item correlation was calculated, with values between 0.2 and 0.4 suggesting that the items are reasonably homogeneous [39]. A good corrected item-total correlation (C-ITC) was considered when values were > 0.3 [40]. Inter-item correlations were also evaluated, and coefficients between 0.15 and 0.5 were considered acceptable [41].

### Ethical considerations

The study was approved by the Ethics and Research Board of the Nursing, Physiotherapy and Medicine Department at the University of Almería (registration number EFM 294/23). In addition, permission to use the instrument was requested from the authors of the original version of the CPRMS. Informed consent to participate was obtained from all of the participants in the study. Participants were informed of the aims of the study, as well as the confidential and anonymous nature of the data, and signed the consent form prior to participation. The Declaration of Helsinki guidelines were followed in this study.

### Data analysis

Results were processed using R (The R Foundation for Statistical Computing, Vienna, Austria) Version 4.3.1 and the R Studio interface (RS Team—RStudio, Inc., Boston, Massachusetts, United States) Version 1.2.5019. Percentages and frequencies were calculated for qualitative variables, measures of central tendency and dispersion for quantitative variables. A 95% confidence interval was established, considering values of *p* < 0.05 as statistically significant.

## Results

### Characteristics of the study participants

The sociodemographic characteristics of the study participants are detailed in Table 1.

**Table 1:** Sociodemographic characteristics of participants

M (SD) | *n* (%)				
Variables	Pilot study *n* = 50	EFA *n* = 120	CFA *n* = 182	Total sample *n* = 352
**Gender**				
Male	11 (22)	27 (22.5)	70 (38.5)	108 (30.7)
Female	39 (78)	93 (77.5)	112 (61.5)	244 (69.3)
**Age (year)**	38.78 (10.53)	28.24 (3.53)	46.33 (8.47)	39.12 (11.13)
**Level of education**				
Bachelor´s degree	24 (48)	65 (54.2)	116 (63.7)	206 (58.5)
Postgraduate course	3 (6.0)	7 (5.8)	11 (6.0)	21 (6.0)
Master´s degree	21 (42)	45 (37.5)	35 (19.2)	100 (28.4)
Doctoral degree	2 (4.0)	3 (2.5)	20 (11)	25 (7.1)
**Professional category**				
Physician	14 (28)	36 (30)	67 (36.8)	117 (33.2)
Nurse	36 (72)	84 (70)	115 (63.2)	235 (66.8)
**Professional experience (years)**	15.42 (10.42)	5.1 (3.1)	21.23 (9.1)	14.51 (10.84)
**Professional experience in critical care and emergency departments**				
< 1 year	4 (8.0)	33 (27.5)	11 (6.0)	48 (13.6)
1 to 3 years	17 (34)	42 (35)	15 (8.2)	74 (21.0)
3-5 years	5 (10)	25 (21.7)	22 (12.1)	52 (14.8)
> 5 years	24 (48)	20 (15.8)	134 (73.6)	178 (50.6)
**Care Units**				
Emergency	15 (30)	50 (41.7)	64 (35.2)	130 (36.9)
Recovery	1 (2.0)	10 (8.3)	4 (2.2)	15 (4.3)
Prehospital emergency	21 (42)	31 (25.8)	71 (39)	123 (34.9)
PICU	-	6 (5)	7 (3.8)	13 (3.7)
Paediatric emergency	1 (2.0)	2 (1.7)	6 (3.3)	9 (2.6)
ICU	11 (22)	18 (15)	21 (11.5)	49 (13.9)
EMS	1 (2.0)	3 (2.5)	9 (4.9)	13 (3.7)

*EMS* Emergency Medical Services, *ICU* Intensive care unit, *PICU* Paediatric intensive care unit

### Content validity

The results of the content validity analysis are presented in Table 2. The s-CVI for the s-CPRMS was 0.88, retaining a total of 24 items with an i-CVI ≥ 0.78 from the original 43 items in the instrument.

**Table 2:** Content validity and reliability of the Spanish version of the cardiopulmonary resuscitation motivation scale (*n* = 50)

CPRMS items	i-CVI (*n* = 13 experts)	Inter-Item Correlation	C-ITC	Ordinal Alpha if item deleted	Scale´s Ordinal Cronbach´s Alpha
1. During the CPR, I try more than usual.	0.92	0.285	0.347	0.901	0.902 I.C=[0.84, 0.95]
2. I make the maximum effort in the CPR because it is important for me to bring back the patient to life.	0.92	0.283	0.386	0.900	
3. I make the maximum effort in the CPR because it is very important for me to save the patient without any harm	1	0.274	0.572	0.896	
*I believe that an attempt to CPR will lead to its success if*					
4. The patient gets a sudden cardio/respiratory arrest.	0.92	0.289	0.259	0.903	
5. The patient with brain damage needs CPR.	0.54	NA	NA	NA	
6. Patient with underlying illnesses (liver, kidney, systemic infection, etc.) requires CPR.	0.69	NA	NA	NA	
7. A patient with cancer needs a CPR.	0.46	NA	NA	NA	
8. The patient needs CPR suddenly	1	0.269	0.676	0.894	
9. The patient often requires CPR.	0.38	NA	NA	NA	
10. The patient has a history of prolonged hypoxia.	0.46	NA	NA	NA	
11. The patient is in the end stage of life.	0.15	NA	NA	NA	
12. The patient is not old.	0.85	0.270	0.658	0.894	
13. The patient has arrested in front of me or my colleagues	0.92	0.279	0.474	0.899	
14. CPR is started immediately after arrest.	1	0.282	0.411	0.900	
15. The patient condition is improving.	0.78	0.284	0.369	0.901	
16. The patient’s conditions are similar to my other successful CPRs in the past.	0.78	0.281	0.424	0.900	
17. I feel very well prepared for doing CPR.	1	0.283	0.385	0.900	
*I’m doing my best in CPR because…*					
18. I believe that God (supernatural power, the supreme being, creator deity) is the main determinant of the time of death.	0.31	NA	NA	NA	
19. I believe that after God (supernatural power, the supreme being, creator deity), I am the main patient assistant.	0.31	NA	NA	NA	
20. I believe that my performance is under divine supervision.	0.31	NA	NA	NA	
21. Of that, the likelihood of understanding my distinctive performance by others is very high.	0.61	NA	NA	NA	
22. Of that, the likelihood of attracting the trust of others (superiors/physicians/patient families) is very high.	0.61	NA	NA	NA	
23. The likelihood of creating opportunities for improving my skills and abilities is very high.	0.78	0.274	0.577	0.896	
24. The likelihood that its success will lead to the satisfaction of the family and companions of the patient is very high.	0.78	0.269	0.676	0.894	
25. It is very likely that its success will lead to job security.	0.54	NA	NA	NA	
26. The likelihood that its success will lead to more respectful behaviours with me is very high.	0.54	NA	NA	NA	
27. Its success makes me feel useful	0.85	0.270	0.654	0.895	
28. Its success makes me feel proud	0.85	0.270	0.659	0.894	
29. I do my moral and humanistic duty	0.92	0.269	0.673	0.894	
30. I do my organizational duty	0.85	0.266	0.733	0.893	
31. It makes me feel better about myself	0.92	0.279	0.467	0.899	
32. Its success makes me experience the pleasurable feeling of patient recovery.	0.78	0.273	0.596	0.896	
33. It causes divine satisfaction.	0.15	NA	NA	NA	
34. It makes me immune to possible legal consequences.	0.31	NA	NA	NA	
35. It reduces the risk of aggression of companions and the family of the patient.	0.46	NA	NA	NA	
36. I must be responsive to patient’s companion.	0.69	NA	NA	NA	
37. I must be accountable to competent authorities.	0.54	NA	NA	NA	
*I’m doing my best in CPR if…*					
38. Having a competent leader in our CPR team	0.92	0.286	0.316	0.902	
39. We have a coordinated team, with competent member	0.92	0.280	0.443	0.899	
40. The CPR environment is tidy and calm, with necessary and adequate facilities and equipment.	0.85	0.280	0.443	0.899	
41. DNAR order is not available for the patient	0.85	0.288	0.284	0.903	
42. There are enough staff to do CPR for the patient	0.78	0.285	0.473	0.899	
43. I do not feel too much fatigue	0.61	NA	NA	NA	

*C-ITC* Corrected Item-Total Correlation, *CPR* Cardiopulmonary Resuscitation, *CPRMS* Cardiopulmonary Resuscitation Motivation Scale, *DNAR* Do Not Attempt Resuscitation, *i-CVI* Content Validity Index-item, *NA* Not applicable

### Pilot study

The reliability results of the pilot study are shown in Table 2. The ordinal alpha was 0.90, indicating excellent reliability for the s-CPRMS. The inter-item correlation coefficients ranged between 0.26 and 0.28, suggesting a fair homogeneity for all items. Most of the items had a C-ITC > 0.3, except items 4 and 41 which were finally retained given their relevance to the measurement construct by agreement among the research team. The ordinal alpha did not increase significantly if any of the items were eliminated; therefore, all items were maintained. The instrument showed adequate temporal stability (mean CCI = 0.701; 95% CI 0.471 to 0.830; *p* < 0.001). Temporal stability was analysed with the retest using the Intraclass Correlation Coefficient (ICC), with values between 0.41 and 0.75 and > 0.75 showing a good and very good correlation, respectively [42].

### Construct validity

**Fig. 1:**
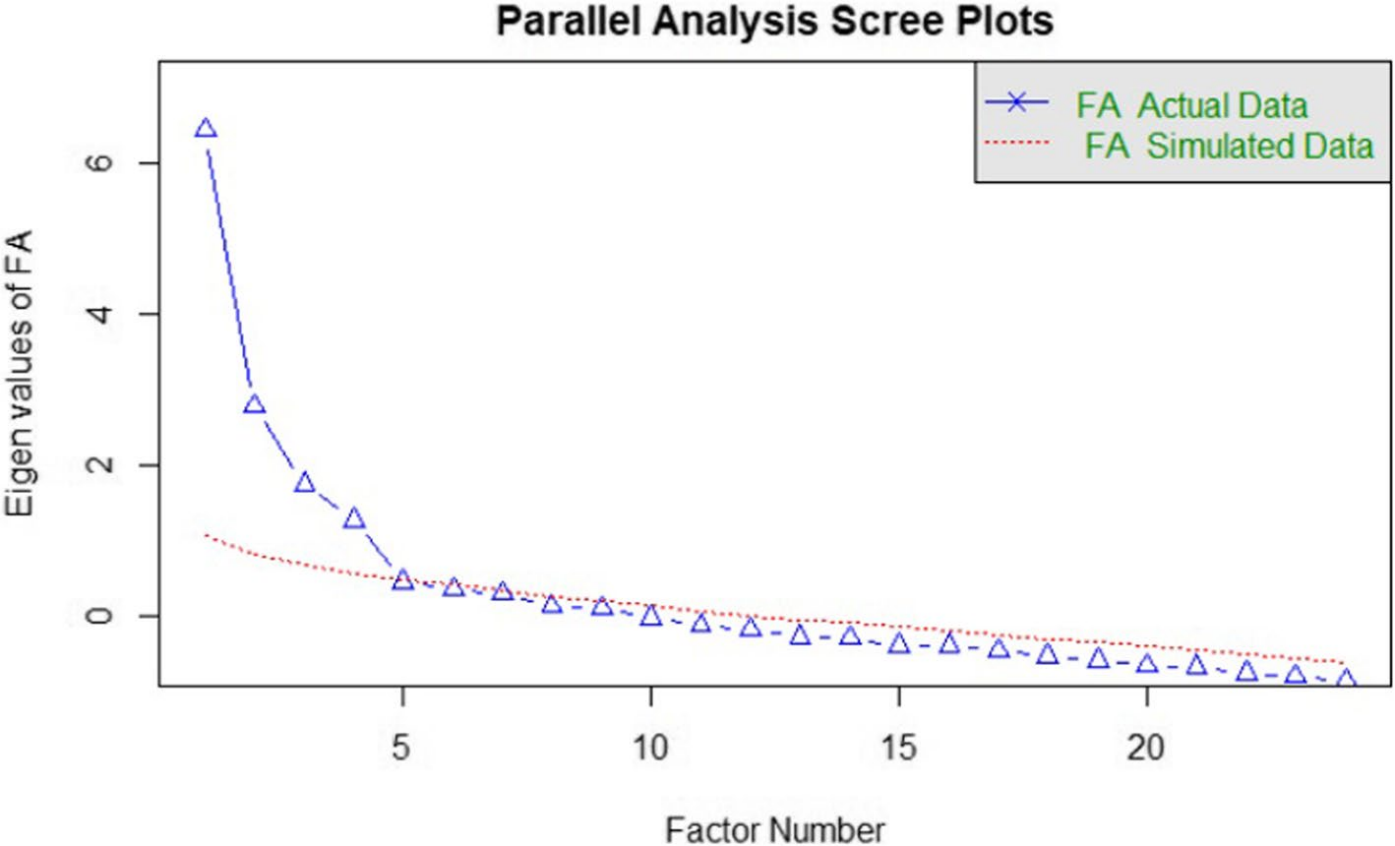
Sree plot

**Fig. 2:**
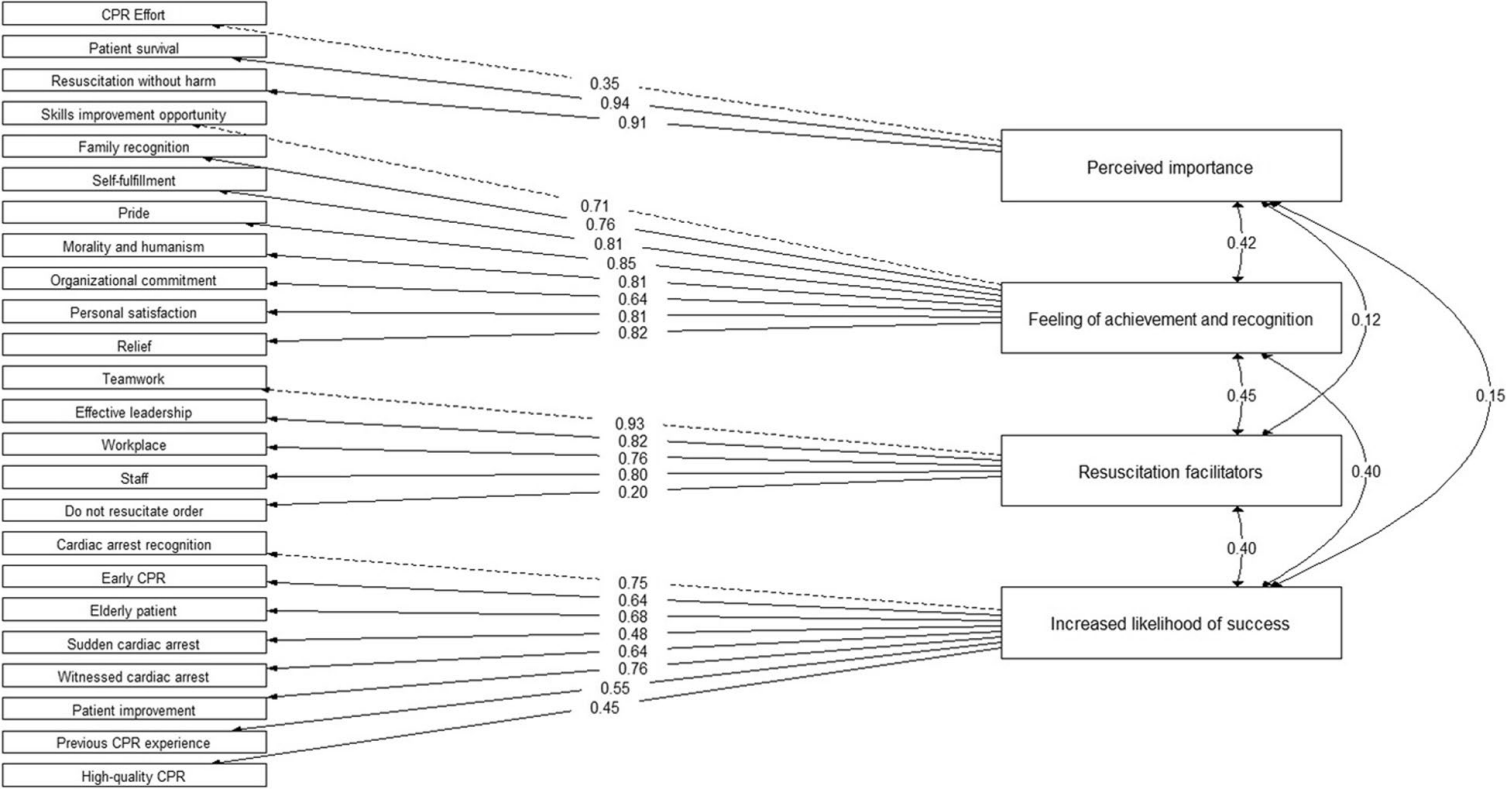
Confirmatory factor analysis model for the dimensional structure of the s-CPRMS

The KMO test was greater than the minimum acceptable (KMO = 0.74) and BST was significant (χ2 (276) = 1262.728; *p* < 0.001); therefore, sampling adequacy was confirmed, and the factorial analysis was appropriate. The scree plot with parallel analysis suggested a four-factor model (Fig. 1). The EFA results showed the s-CPRMS was distributed into four factors that explained the 58% of the total variance (Table 3). Factor loading was > 0.3 for all the items. After EFA results, a CFA was carried out to confirm the latent structure of the four-factor model fit proposed for the s-CPRMS, with values of the goodness of fit indices indicating an adequate fit for the model extracted: χ2 (246) = 402.240; *p* < 0.001, SRMR = 0.086, RMSEA = 0.059 (95% IC = 0.046–0.071), TLI = 0.965, CFI = 0.969, GFI = 0.990. Therefore, all of the 24 items were retained in the final s-CPRMS (Fig. 2) structured into the following four factors: (1) Perceived importance (2) Feeling of achievement and recognition (3) Resuscitation facilitators and (4) Increased likelihood of success.

**Table 3:** Exploratory factor analysis for the Spanish version of the cardiopulmonary resuscitation motivation scale (*n* = 120)

s-CPRMS	F1	F2	F3	F4	h2	u2	com
**Factor 1. Perceived importance**							
I1. During the CPR, I try more than usual.	0.76				0.70	0.29	1.20
I2. I make the maximum effort in the CPR because it is important for me to bring back the patient to life.	0.91				0.82	0.17	1.00
I3. I make the maximum effort in the CPR because it is very important for me to save the patient without any harm.	0.76				0.66	0.33	1.25
**Factor 2. Feeling of achievement and recognition**							
I23. The likelihood of creating opportunities for improving my skills and abilities is very high.		0.54			0.39	0.60	1.24
I24. The likelihood that its success will lead to the satisfaction of the family and companions of the patient is very high.		0.55			0.39	0.60	1.44
I27. Its success makes me feel useful.		0.81			0.61	0.38	1.06
I28. Its success makes me feel proud.		0.87			0.76	0.23	1.05
I29. I do my moral and humanistic duty.		0.84			0.72	0.27	1.04
I30. I do my organizational duty.		0.79			0.69	0.30	1.03
I31. It makes me feel better about myself.		0.91			0.83	0.16	1.04
I32. Its success makes me experience the pleasurable feeling of patient recovery.		0.84			0.76	0.23	1.05
**Factor 3. Resuscitation facilitators**							
I38. Having a competent leader in our CPR team.			0.91		0.79	0.20	1.04
I39. We have a coordinated team, with competent member.			0.92		0.95	0.04	1.04
I40. The CPR environment is tidy and calm, with necessary and adequate facilities and equipment.			0.87		0.74	0.25	1.00
I41. DNAR order is not available for the patient.			0.38		0.16	0.83	1.23
I42.There are enough staff to do CPR for the patient.			0.59		0.51	0.48	1.30
**Factor 4. Increased likelihood of success**							
I4. The patient gets a sudden cardio/respiratory arrest.				0.37	0.20	0.79	1.40
I8. The patient needs CPR suddenly (for example, drowning, shock, poisoning, etc.).				0.63	0.50	0.49	1.59
I12. The patient is not old.				0.42	0.27	0.72	2.00
I13. The patient has arrested in front of me or my colleagues.				0.75	0.55	0.44	1.06
I14. CPR is started immediately after arrest.				0.49	0.52	0.47	2.53
I15. The patient’s condition is improving.				0.66	0.49	0.50	1.20
I16. The patient’s conditions are similar to my other successful CPRs in the past.				0.71	0.55	0.44	1.10
I17. I feel very well prepared for doing CPR.				0.34	0.16	0.83	1.39
% of variance	0.22	0.14	0.12	0.10			
% of accumulated variance	0.22	0.36	0.48	0.58			

*CPR* Cardiopulmonary Resuscitation, *DNAR* Do Not Attempt Resuscitation, *s-CPRMS* Spanish version of the Cardiopulmonary Resuscitation Motivation Scale

h2 = communality of the item, u2 = uniqueness of the item, com = Hoffmann’s item of complexity

### Reliability

The ordinal alpha for the total s-CPRMS was 0.847, ranging from 0.796 to 0.923 for the factors which indicates and excellent reliability for the instrument (Table 4). The C-ITC was higher than 0.30 for all the items. An increased ordinal alpha coefficient when removing items 1 and 41 was observed. Nevertheless, satisfying the minimum standard for alpha > 0.70, the research team agreed to retain them in the final version of the instrument given the relevance pointed out by the experts.

**Table 4:** Reliability of the final version of the s-CPRMS (*n* = 182)

	Ordinal Alpha if item Deleted	C-ITC	Scale’s Ordinal Alpha
**F1. Perceived importance**			
Item 1.	**0.857**	0.506	0.796
Item 2.	0.639	0.715	
Item 3.	0.645	0.710	
**F2. Feeling of achievement and recognition**			
Item 23.	0.918	0.717	0.923
Item 24.	0.915	0.761	
Item 27.	0.911	0.808	
Item 28.	0.905	0.883	
Item 29.	0.911	0.823	
Item 30.	**0.924**	0.657	
Item 31.	0.910	0.829	
Item 32.	0.913	0.777	
**F3. Resuscitation facilitators**			
Item 38.	0.789	0.691	0.836
Item 39.	0.758	0.796	
Item 40.	0.763	0.780	
Item 41.	**0.889**	0.304	
Item 42.	0.796	0.665	
**F4. Increased likelihood of success**			
Item 4.	0.827	0.449	0.832
Item 8.	0.805	0.617	
Item 12.	0.814	0.543	
Item 13.	0.791	0.716	
Item 14.	0.800	0.649	
Item 15.	0.804	0.620	
Item 16.	0.817	0.525	
Item 17.	**0.838**	0.361	
**Total s-CPRMS**			0.847

## Discussion

This study aimed to translate and cross-culturally adapt the Cardiopulmonary Resuscitation Motivation Scale into Spanish and assess its psychometric properties to assess CPR motivation in critical care and emergency care teams in the Spanish context.

The translation and adaptation of the instrument into Spanish was carried out through a rigorous process to ensure cross-cultural equivalence of content, semantic, technical, criterion, and conceptual with the original version [32], showing appropriate content validity for the total and each of the items of the s-CPRMS after expert evaluation. The results of the pilot study revealed adequate internal consistency and temporal stability for the s-CPRMS, with alpha values for the total scale similar to those obtained by [26] indicating excellent reliability. However, unlike the original study, in this research the reliability analysis was not performed using Cronbach’s alpha but through the ordinal Alpha due to the growing literature supporting the adequacy of this coefficient for the calculation of reliability when the data are ordinal [43, 44].

Exploratory factor analysis revealed a four-factor structure for the s-CPRMS that matched the factors ‘resuscitation facilitators’, ‘high chances of success’, ‘perceived importance’, ‘feeling of achievement’ and ‘recognition and appreciation’ originally described in the instrument [26]. Factor loading results suggested including the items from the ‘feeling of achievement’ and ‘recognition and appreciation’ of the CPRMS into a single factor entitled ‘feeling of achievement and recognition’ for the s-CPRMS. In contrast, the factors ‘low chances of success’, ‘accountability’ and ‘beliefs’ were removed from the structure of the s-CPRMS, retaining only 24 of the original 43 items of the original instrument. This could be due to religious and cultural differences between the study countries [45], supporting the importance of adapting and validating instruments that allow to compare outcomes between different countries, cultural groups, as well as, health plans and policies and make meaningful contributions to improve CPR quality and patient health outcomes [46].

The CFA results showed satisfactory values of the goodness of fit indexes for the four-factor model fit extracted for the s-CPRMS. Previous studies pointed out the likelihood of success as one of the most important motivational factors in CPR [47]. However, a weak correlation between the factors ‘perceived importance’ and ‘high chance of success’ was observed in this study. At early stages of cardiac arrest is difficult to accurately estimate the real chances of success of CPR in spite of knowing predictors associated with better survival and neurological outcomes [2, 48]. Then it might be that healthcare professionals, in attempt to provide a prompt response, focus their efforts on performing high-quality CPR as the main component related to improved outcomes after cardiac arrest more than on likelihood of success to prevent patient from potential harm.

Regarding the reliability of the final version of the s-CPRMS, the results showed adequate reliability (internal consistency) with values of 0.84 for the total scale and a range of 0.79–0.92 for the factors. An improvement in the reliability coefficient was observed when deleting items 4 and 41 related ‘witnessed cardiac arrest’ and ‘do not resuscitation order’, respectively. However, both were retained in the final version of the s-CPRMS mainly for the following reasons. On the one hand, we considered their outstanding relevance as stated by the expert panel and the literature in the measurement of CPR motivation as a phenomenon of study interest. On the other hand, some authors suggest that alpha values higher than 0.90 could indicate redundancy or duplication, being preferable values between 0.80 and 0.90 [30, 33]. Thus, the results of this research demonstrate the use of the s-CPRMS as a valid and reliable instrument for analyzing the motivation of critical care and emergency care physicians and nurses in the Spanish healthcare context.

### Limitations and future research

This study had some limitations to consider. Despite guaranteeing the minimum recommended for the EFA and CFA, a larger sample size would have been appropriate. Participants were recruited by convenience; therefore, considering the possible differences between groups and countries, the results obtained should not be extrapolated to other samples or study contexts. Consequently, more research is needed in other geographical and cultural contexts to allow comparison in the future. Also, studies focusing on the mediating effects of motivation in high-quality CPR performance and patient outcomes are imperative.

## Conclusions

The Spanish version of the Cardiopulmonary Resuscitation Motivation Scale is a valid and reliable instrument to measure CPR motivation of medical and nursing health care professionals in the Spanish health care context. Knowing the motivations of rescuers in CPR can be useful for identifying their individual motivations and personalizing training, preventing professional burnout, as well as improving team collaboration and communication, strengthening effective and coordinated response to emergency situations. Thus, the application of the instrument can lead to the creation of potentially effective strategies to motivate resuscitators in performing high-quality CPR with outstanding patient outcomes, contributing to improved quality of care and health personnel performance during CPR.

## Data Availability

The datasets used and/or analysed during the current study are available from the corresponding author on reasonable request.

## References

[1] O’Leary A, Butler P, Fine JR. Dedicated chest compressor team: a quality improvement initiative to improve chest compression performance at in-hospital cardiac arrest events through quarterly training. Resusc Plus. 2023;13:100361. 10.1016/j.resplu.2023.100361. 36798488 10.1016/j.resplu.2023.100361 PMC9926014

[2] Penketh J, Nolan JP. In-hospital cardiac arrest: the state of the art. Crit Care. 2022;26(1):376. 10.1186/s13054-022-04247-y. 36474215 10.1186/s13054-022-04247-y PMC9724368

[3] Perkins GD, Graesner JT, Semeraro F, Olasveengen T, Soar J, Lott C, Van de Voorde P, Madar J, Zideman D, Mentzelopoulos S, Bossaert L, Greif R, Monsieurs K, Svavarsdóttir H, Nolan JP, European Resuscitation Council Guideline Collaborators. European Resuscitation Council guidelines 2021: executive summary. Resuscitation. 2021;161:1–60. 10.1016/j.resuscitation.2021.02.003. 33773824 10.1016/j.resuscitation.2021.02.003

[4] Sumner BD, Hahn CW. Prognosis of cardiac arrest-peri-arrest and post-arrest considerations. Emerg Med Clin North Am. 2023;41(3):601–16. 10.1016/j.emc.2023.03.008. 37391253 10.1016/j.emc.2023.03.008

[5] Hernando ASC, Nieves-Alonso JM, Mjertan A, Martínez DG, Roca AP. In-hospital cardiorespiratory arrest: incidence, prognostic factors and outcomes. Span J Anesthesiolo Resusc. 2023. 10.1016/j.redare.2022.06.006.

[6] Vazquez AR, Sudhir A. Cardiac arrest as a public health issue. Emerg Med Clin North Am. 2023;41(3):405–11. 10.1016/j.emc.2023.05.003. Epub 2023 Jun 5 PMID: 37391241. 37391241 10.1016/j.emc.2023.05.003

[7] DiLibero J, Misto K. Outcomes of in-hospital cardiac arrest: a review of the evidence. Crit Care Nurs Clin North Am. 2021;33(3):343–56. 10.1016/j.cnc.2021.05.009. 34340795 10.1016/j.cnc.2021.05.009

[8] Andersen LW, Holmberg MJ, Berg KM, Donnino MW, Granfeldt A. In-hospital cardiac arrest: a review. JAMA. 2019;321(12):1200–10. 10.1001/jama.2019.1696. 30912843 10.1001/jama.2019.1696 PMC6482460

[9] Colwill M, Somerville C, Lindberg E, Williams C, Bryan J, Welman T. Cardiopulmonary resuscitation on television: are we miseducating the public? Postgrad Med J. 2018;94(1108):71–5. 10.1136/postgradmedj-2017-135122. 28993522 10.1136/postgradmedj-2017-135122

[10] Picard C, Drew R, Norris CM, O’Dochartaigh D, Burnett C, Keddie C, Douma MJ. Cardiac arrest quality improvement: a single-center evaluation of resuscitations using defibrillator, feedback device, and survey data. J Emerg Nurs. 2022;48(2):224-e2328. 10.1016/j.jen.2021.11.005. 35249668 10.1016/j.jen.2021.11.005

[11] Mitchell OJL, Shi X, Abella BS, Girotra S. Mechanical cardiopulmonary resuscitation during in-hospital cardiac arrest. J Am Heart Assoc. 2023;12(7):e027726. 10.1161/JAHA.122.027726. 36942764 10.1161/JAHA.122.027726 PMC10122908

[12] Andersson A, Arctaedius I, Cronberg T, Levin H, Nielsen N, Friberg H, Lybeck A. In-hospital versus out-of-hospital cardiac arrest: characteristics and outcomes in patients admitted to intensive care after return of spontaneous circulation. Resuscitation. 2022;176:1–8. 10.1016/j.resuscitation.2022.04.023. 35490935 10.1016/j.resuscitation.2022.04.023

[13] Gerecht RB, Nable JV. Out-of-hospital cardiac arrest. Emerg Med Clin North Am. 2023;41(3):433–53. 10.1016/j.emc.2023.03.002. 37391243 10.1016/j.emc.2023.03.002

[14] Torsy T, Deswarte W, Karlberg Traav M, Beeckman D. Effect of a dynamic mattress on chest compression quality during cardiopulmonary resuscitation. Nurs Crit Care. 2022;27(2):275–81. 10.1111/nicc.12631. 33884701 10.1111/nicc.12631

[15] Bishop R, Joy B, Moore-Clingenpeel M, Maa T. Automated audiovisual feedback in cardiopulmonary resuscitation training: improving skills in pediatric intensive care nurses. Crit Care Nurs. 2018;38(5):59–66. 10.4037/ccn2018490. 10.4037/ccn2018490 30275064

[16] Cogan ES, Thomas LMB. Improving CPR quality through high-performance resuscitation team training. Nursing. 2022;52(9):57–9. 10.1097/01.NURSE.0000854016.95250.ac. 36006755 10.1097/01.NURSE.0000854016.95250.ac

[17] Lim WY, Ong J, Ong S, Teo LM, Fook-Chong S, Ho VK. Rapid degradation of psychomotor memory causes poor quality chest compressions in frequent cardiopulmonary resuscitation providers and feedback devices can only help to a limited degree: a crossover simulation study. Med (Baltim). 2021;100(8):e23927. 10.1097/MD.0000000000023927. 10.1097/MD.0000000000023927 PMC7909212 33663043

[18] Berdida DJE. Resilience and academic motivation’s mediation effects in nursing students’ academic stress and self-directed learning: a multicenter cross-sectional study. Nurse Educ Pract. 2023;69:103639. 10.1016/j.nepr.2023.103639. 37060734 10.1016/j.nepr.2023.103639

[19] Fong CJ, Patall EA, Snyder KE, Hoff MA, Jones SJ, Zuniga-Ortega RE. Academic underachievement and self-conceptual, motivational, and self-regulatory factors: a meta-analytic review of 80 years of research. Educ Res Rev. 2023;100566:100566. 10.1016/j.edurev.2023.100566.

[20] Hsu HP, Guo JL, Lin FH, Chen SF, Chuang CP, Huang CM. Effect of involvement and motivation on self-learning: evaluating a mobile e-learning program for nurses caring for women with gynecologic cancer. Nurse Educ Pract. 2023;67:103558. 10.1016/j.nepr.2023.103558. 36738527 10.1016/j.nepr.2023.103558

[21] Kuzovlev A, Monsieurs KG, Gilfoyle E, Finn J, Greif R, Education Implementation, Teams Task Force of the International Liaison Committee on Resuscitation. The effect of team and leadership training of advanced life support providers on patient outcomes: a systematic review. Resuscitation. 2021;160:126–39. 10.1016/j.resuscitation.2021.01.020. 33556422 10.1016/j.resuscitation.2021.01.020

[22] Mota S. Resuscitation quality improvement: improving clinicians’ performance. AACN Adv Crit Care. 2023;34(3):182–8. 10.4037/aacnacc2023833. 37644632 10.4037/aacnacc2023833

[23] Marks S, Shaffer L, Zehnder D, Aeh D, Prall DM. Under pressure: what individual characteristics lead to performance of high-quality chest compressions during CPR practice sessions? Resusc Plus. 2023;14:100380. 10.1016/j.resplu.2023.100380. 37035444 10.1016/j.resplu.2023.100380 PMC10074238

[24] Tramèr L, Becker C, Schumacher C, Beck K, Tschan F, Semmer NK, Hochstrasser S, Marsch S, Hunziker S. Association of self-esteem, personality, stress and gender with performance of a resuscitation team: a simulation-based study. PLoS One. 2020;15(5):e0233155. 10.1371/journal.pone.0233155. 32407382 10.1371/journal.pone.0233155 PMC7224528

[25] Bushuven S, Bansbach J, Bentele M, Trifunovic-Koenig M, Bentele S, Gerber B, Hagen F, Friess C, Fischer MR. Overconfidence effects and learning motivation refreshing BLS: an observational questionnaire study. Resusc Plus. 2023;14:100369. 10.1016/j.resplu.2023.100369. 36935817 10.1016/j.resplu.2023.100369 PMC10020094

[26] Assarroudi A, Heshmati Nabavi F, Ebadi A. Motivation for cardiopulmonary resuscitation: scale development and psychometric analysis. Int Emerg Nurs. 2019;45:43–9. 10.1016/j.ienj.2019.02.005. 31047853 10.1016/j.ienj.2019.02.005

[27] Najafi M, Yadollahi S, Maghami M, Azizi-Fini I. Nurses’ motivation for performing cardiopulmonary resuscitation: a cross-sectional study. BMC Nurs. 2024;23(1):181. 10.1186/s12912-024-01853-9. 38486281 10.1186/s12912-024-01853-9 PMC10941359

[28] Hertzog MA. Considerations in determining sample size for pilot studies. Res Nurs Health. 2008;31(2):180–91. 10.1002/nur.20247. 18183564 10.1002/nur.20247

[29] Gorsuch RL. Factor analysis. 2nd ed. Hillsdale: Erlbaum; 1983.

[30] Streiner DL, Norman GR, Cairney J. Health measurement scales: a practical guide to their development and use. 5th ed. 2015. 10.1093/med/9780199685219.001.0001.

[31] Polit D, Beck C. Essentials of nursing research: appraising evidence for nursing practice. 10th ed. Philadelphia, Lippincott Williams & Wilkins; 2021.

[32] Squires A, Aiken LH, van den Heede K, Sermeus W, Bruyneel L, Lindqvist R, Schoonhoven L, Stromseng I, Busse R, Brzostek T, Ensio A, Moreno-Casbas M, Rafferty AM, Schubert M, Zikos D, Matthews A. A systematic survey instrument translation process for multi-country, comparative health workforce studies. Int J Nurs Stud. 2013;50(2):264–73. 10.1016/j.ijnurstu.2012.02.015. 22445444 10.1016/j.ijnurstu.2012.02.015 PMC3395768

[33] Polit DF, Beck CT. Nursing research: generating and assessing evidence for nursing practice. 11th ed. Philadelphia, Wolters Kluwer; 2021.

[34] Brown TA. Confirmatory factor analysis for applied research. New York: Guilford Press; 2006.

[35] Hu LT, Bentler PM. Cutoff criteria for fit indexes in covariance structure analysis: conventional criteria versus new alternatives. Struct Equ Model –Multidiscip J. 1999;6(1):1–55.

[36] Espinoza SC, Novoa-Muñoz F. Ventajas Del alfa ordinal respecto Al Alfa De Cronbach ilustradas con la encuesta AUDIT-OMS [Advantages of ordinal alpha versus Cronbach’s alpha, illustrated using the WHO AUDIT testVantagens do alfa ordinal em relação Ao Alfa De Cronbach verificadas na pesquisa AUDIT-OMS]. Rev Panam Salud Publ. 2018;42:e65. 10.26633/RPSP.2018.65. 10.26633/RPSP.2018.65 PMC6398318 31093093

[37] Gadermann AM, Guhn M, Zumbo D. Estimating ordinal reliability for likert-tipe and ordinal item response data: a conceptual, empirical, and practical guide. Practical Assess Res Evaluation. 2012;17(3):1–13.

[38] McNeish D. Thanks coefficient alpha, we’ll take it from here. Psychol Methods. 2018;23:412–33. 28557467 10.1037/met0000144

[39] Cohen RJ, Swerdlik ME. Psychological testing and assessment: an introduction to tests and measurement. 6th ed. New York: McGraw-Hill; 2005.

[40] Kline P. The handbook of psychological testing. London: Routledge; 1999.

[41] Clark LA, Watson D. Constructing validity: basic issues in objective scale development. PsycholAssess. 1995;7(3):309–19. 10.1037/1040-3590.7.3.309.

[42] Fleiss JL. The design and analysis of clinical experiments. New York: John Wiley & Sons; 1986.

[43] Viladrich C, Angulo-Brunet A, Doval E. Un viaje alrededor de alfa y omega para estimar la fiabilidad de consistencia interna. Anales De Psicología. 2017;33(3):755–82.

[44] Vizioli N, Pagano A. De alfa a omega: Estimación de la confiabilidad ordinal. Una guía práctica. Revista Costarricense De Psicología. 2022;41(2):119–36. 10.22544/rcps.v41i02.02.

[45] Arnault DS. Defining and theorizing about culture: the evolution of the cultural determinants of help-seeking, revised. Nurs Res. 2018;67(2):161–8. 10.1097/NNR.0000000000000264. 29489636 10.1097/NNR.0000000000000264 PMC7439772

[46] Hunt SM, Alonso J, Bucquet D, Niero M, Wiklund I, McKenna S. Cross-cultural adaptation of health measures. European Group for Health Management and Quality of Life Assessment. Health Policy. 1991;19(1):33–44. 10.1016/0168-8510(91)90072-6. 10117390 10.1016/0168-8510(91)90072-6

[47] Assarroudi A, Heshmati Nabavi F, Ebadi A, Esmaily H. Professional rescuers’ experiences of motivation for cardiopulmonary resuscitation: a qualitative study. Nurs Health Sci. 2017;19(2):237–43. 10.1111/nhs.12336. 28247467 10.1111/nhs.12336

[48] Marinšek M, Sinkovič A, Šuran D. Neurological outcome in patients after successful resuscitation in out-of-hospital settings. Bosn J Basic Med Sci. 2020;20(3):389–95. 10.17305/bjbms.2020.4623. 32156250 10.17305/bjbms.2020.4623 PMC7416179

